# The influence of women’s preferences and actual mode of delivery on post-traumatic stress symptoms following childbirth: a population-based, longitudinal study

**DOI:** 10.1186/1471-2393-14-191

**Published:** 2014-06-05

**Authors:** Susan Garthus-Niegel, Tilmann von Soest, Cecilie Knoph, Tone Breines Simonsen, Leila Torgersen, Malin Eberhard-Gran

**Affiliations:** 1 HØKH, Research Centre, Akershus University Hospital, Lørenskog, Norway; 2 Department of Psychosomatics and Health Behavior, Norwegian Institute of Public Health, Oslo, Norway; 3 Institute and Outpatient Clinics of Occupational and Social Medicine, TU Dresden, Faculty of Medicine, Fetscherstr. 74, Dresden 01307, Germany; 4 Department of Childhood, Development and Cultural Diversity, Norwegian Institute of Public Health, Oslo, Norway; 5 Department of Psychology, University of Oslo, Oslo, Norway; 6 Division of Mental Health Services, Akershus University Hospital, Lørenskog, Norway; 7 Department of Obstetrics and Gynaecology, Akershus University Hospital, Lørenskog, Norway

**Keywords:** Post-traumatic stress symptoms following childbirth, Mode of delivery, Mismatch, Akershus birth cohort

## Abstract

**Background:**

This study aimed to examine whether a mismatch between a woman’s preferred and actual mode of delivery increases the risk of post-traumatic stress symptoms after childbirth.

**Methods:**

The study sample consisted of 1,700 women scheduled to give birth between 2009 and 2010 at Akershus University Hospital, Norway. Questionnaire data from pregnancy weeks 17 and 32 and from 8 weeks postpartum were used along with data obtained from hospital birth records. Post-traumatic stress symptoms were measured with the Impact of Event Scale. Based on the women’s preferred and actual mode of delivery, four groups were established: Match 1 (no preference for cesarean section, no elective cesarean section, N = 1,493); Match 2 (preference for cesarean section, elective cesarean section, N = 53); Mismatch 1 (no preference for cesarean section, elective cesarean section, N = 42); and Mismatch 2 (preference for cesarean section, no elective cesarean section, N = 112). Analysis of variance (ANOVA) and analysis of covariance (ANCOVA) were conducted to examine whether the level of post-traumatic stress symptoms differed significantly among these four groups.

**Results:**

Examining differences for all four groups, ANOVA yielded significant overall group differences (F = 11.96, p < 0.001). However, Bonferroni post-hoc tests found significantly higher levels of post-traumatic stress symptoms only in Mismatch 2 compared to Match 1. This difference could be partly explained by a number of risk factors, particularly psychological risk factors such as fear of childbirth, depression, and anxiety.

**Conclusions:**

The results suggest increased post-traumatic stress symptoms in women who preferred delivery by cesarean section but delivered vaginally compared to women who both preferred vaginal delivery and delivered vaginally. In psychologically vulnerable women, such mismatch may threaten their physical integrity and, in turn, result in post-traumatic stress symptoms. These women, who often fear childbirth, may prefer a cesarean section even though vaginal delivery is usually the best option in the absence of medical indications. To avoid potential trauma, fear of childbirth and maternal requests for a cesarean section should be taken seriously and responded to adequately.

## Background

Up to one-third of women view their labor and delivery as traumatic. An estimated 2-6% of women experience the full constellation of symptoms of post-traumatic stress disorder (PTSD) and qualify for a clinical diagnosis [1]. The prevalence of these symptoms has typically been measured within the first six months postpartum, but there is evidence suggesting the potential longevity of post-traumatic stress responses in some women [2]. Higher levels of post-traumatic stress symptoms are associated with an increased likelihood of not having additional children or of delaying a subsequent pregnancy [3,4]. In addition, it has been suggested that a new pregnancy has the potential to reactivate post-traumatic stress symptoms [1]. Knowledge about risk factors of post-traumatic stress symptoms following childbirth is, therefore, of great importance, and may help identify mothers who may need intervention.

Previous studies identified a number of psychosocial and medical risk factors, such as depression and anxiety, and infant and maternal perinatal complications [5]. Also, the mode of delivery has repeatedly been shown to be a risk factor for post-traumatic stress symptoms. In particular, a number of studies found an increased risk of post-traumatic stress responses after an emergency cesarean section (CS) [6-8]. However, a vaginal delivery or an elective CS may also result in post-traumatic stress symptoms. For instance, in Søderquist and colleagues’ study, most women with a PTSD symptom profile were found among those who had a normal vaginal delivery [7]. This may reflect that, beyond the absence or presence of obstetric interventions such as an emergency CS, both psychological vulnerability and the subjective birth experience may be crucial in the development of post-traumatic stress symptoms [7,9].

Similarly, another potential risk factor for a delivery to be perceived as traumatic may be a *mismatch* between the women’s expectations or preferences and the actual birth experience [1,10]. Although comparatively few women request a CS [11,12], some do so because of psychological reasons and fear of childbirth [12,13]. If the fear is severe and a woman is forced to deliver vaginally, she might experience the situation as traumatic. Soet and colleagues even suggest that “Either way, when the childbirth does not fit with expectations, it may be more likely to be perceived as traumatic” [10]. Lazarus emphasizes the role of goal congruence or incongruence (mismatch) for our emotions: If conditions are congruent with what a person wishes, a positively toned emotion is likely to be aroused. On the other hand, if conditions thwart the person’s wishes, a negatively toned emotion is likely to result [14].

Regarding mode of delivery, two potential mismatches are conceivable; some women would prefer a vaginal delivery, but need to deliver by CS, e.g. for medical reasons [15], whereas others would prefer an elective CS, but their wish is not granted and they have to deliver vaginally (compared with other developed countries, Norway has a relatively low rate [16.2%] of CS) [16,17].

To our knowledge, no previous study has evaluated whether a *mismatch* between the preferred and actual mode of delivery, rather than the delivery mode in and of itself, increases the risk of developing post-traumatic stress symptoms following childbirth.

### Aim

In this prospective cohort study we followed women from pregnancy through eight weeks post-partum. Our aim was to examine the following research questions:

Does a mismatch between the preferred and actual mode of delivery increase the risk of developing post-traumatic stress symptoms following childbirth?

a) Regarding such risks, is there a difference depending on the type of mismatch: preference for vaginal delivery but delivery by elective CS vs. preference for CS but vaginal delivery?

b) If an increased risk is found for one or both kinds of mismatch, are there putative risk factors that may account for such an association?

## Methods

### Design and participants

The study sample was drawn from the Akershus Birth Cohort (ABC), which targeted all women scheduled to give birth at Akershus University Hospital, located near Oslo, Norway, which serves approximately 350,000 people from both urban and rural areas. On average, 4,200 women give birth at the hospital’s maternity ward each year.

Recruitment took place from November 2008 to April 2010. Mothers were recruited for the study during their routine fetal ultrasound examination, which is performed around gestational week 17. Of the eligible women (i.e. those able to complete a questionnaire in Norwegian), 80% (N = 3,752) agreed to participate and returned the first questionnaire. Participants also completed questionnaires at pregnancy week 32 and 8 weeks postpartum. The number of eligible women dropped somewhat at pregnancy week 32 and 8 weeks postpartum because some women had moved or were withdrawn from the study because of severe complications (N = 261). Response rates were 81% (2,936 out of 3,620) and 79% (2,217 out of 2806) at pregnancy week 32 and 8 weeks postpartum, respectively. 1,984 women completed all 3 questionnaires. For the present study, we used information from all three questionnaires as well as information from the hospital’s birth records. The birth record is completed by the hospital staff and contains sociodemographic and medical information about the mother, child, pregnancy, and birth. We excluded women who underwent an emergency CS (N = 185) as these procedures are contingent on ad hoc labor complications and not on a disregard of a woman’s preference concerning mode of delivery.

The ABC study obtained ethical approval from the Regional Committees for Medical and Health Research Ethics (approval number S-08013a), and all participants provided written informed consent.

### Measures

#### Post-traumatic stress symptoms following childbirth

The Impact of Event Scale [18] was used to measure post-traumatic stress symptoms 8 weeks postpartum. The Impact of Event Scale is a self-rating scale that measures symptoms of intrusion and avoidance. The scale has four response categories with the following weightings: 0 = not at all, 1 = rarely, 3 = sometimes, and 5 = often. Higher scores reflect a higher degree of post-traumatic stress, and a score above 34 on the Impact of Event Scale has been suggested to indicate that a PTSD condition is likely to be present [19].

#### Preference and actual mode of delivery

“Preference for a CS” was based on the following question during pregnancy week 32: “If I could choose, I would rather deliver by CS.” The answers were coded as: yes (“highly agree”, “agree”) or no (“disagree”, “highly disagree”). The reason for the respective preference was not assessed.

Information on delivery by elective CS was obtained from the hospital’s birth record. The term elective CS includes cesarean deliveries planned 8 hours or more before delivery.

#### Putative risk factors

##### Psychological factors

During pregnancy week 17, the women reported whether they had been involved in or had experienced a dramatic and terrifying event at any time in their life. If they answered affirmatively, they then reported whether or not they had experienced eight potential symptoms related to that event during the past month. The symptoms were based on the questions regarding PTSD included in the Mini-International Neuropsychiatric Interview (M.I.N.I.). The M.I.N.I, designed for epidemiological studies and clinical trials, is a short, structured, clinical interview that enables researchers to make diagnoses of psychiatric disorders according to the DSM-IV or ICD-10 [20]. The symptoms that we measured were “During the last month I…: (1) “re-experienced the event (e.g., in dreams, nightmares, intense memories, or flashbacks);” (2) “avoided thinking or talking about the event”; (3) “had problems remembering the event”; (4) “felt distant”; (5) “had trouble sleeping”; (6) “had trouble concentrating”; (7) “was nervous”; and (8) “was considerably disturbed by the event in my work and in social activities”. Participants were given scores for symptoms that were present, which resulted in a symptom score ranging from 0 (no symptoms) to 8 (maximum number of symptoms).

History of sexual abuse was assessed with an adapted version of the Abuse Assessment Screen [21] at 8 weeks postpartum. The questions were: “Have you ever been coerced or forced into sexual activities?” The answers were coded as yes (“yes, exercised power”, “yes, coerced”, or “yes, raped”) or no (“no, never”).

Fear of childbirth was assessed during pregnancy week 32 using the Wijma Delivery Expectancy/Experience Questionnaire (W-DEQ, version A) [22]. This is the most frequently used instrument to measure fear of childbirth. The W-DEQ, version A, measures fear of childbirth as operationalized by the cognitive appraisal of the approaching delivery. The 33-item rating scale has six response categories ranging from 0 to 5. Summary scores can have a minimum score of 0 and a maximum score of 165, with higher scores reflecting a greater degree of fear of childbirth.

Symptoms of depression during the past week were measured using the Edinburgh Postnatal Depression Scale [23] during pregnancy week 32. The Edinburgh Postnatal Depression Scale is a 10-item self-rating scale designed to identify postnatal depression. The scale has four response categories ranging from 0 to 3; thus, the total scores can range from 0 to 30. Higher scores reflect higher levels of depression. The scale has been validated for use in pregnancy as well as with non-postnatal mothers [24,25].

Ten items from the Hopkins Symptom Check List were used to evaluate anxiety symptoms during the previous week during pregnancy week 32. The Hopkins Symptom Check List is a widely used self-rating scale, and the first 10 items comprise the anxiety score (SCL-anxiety). The scale has four response categories ranging from 1 to 4. Consequently, total scores range from 10 to 40, with higher scores indicating higher levels of anxiety [26,27].

##### Personality

Personality was assessed with a Norwegian version of the Mini-IPIP scale [28]. The Mini-IPIP, a 20-item short form of the 50-item International Personality Item Pool-Five Factor Model measure [29], was developed and validated across five studies. The results show that the Mini-IPIP is a psychometrically acceptable and practically useful short measure of the Big Five factors of personality, i.e. Extraversion, Agreeableness, Conscientiousness, Neuroticism, and Intellect/Imagination. Each personality factor is measured with four items on a 5 point Likert scale, and scores can range from 5 to 20 for each factor.

##### Somatic and demographic factors

Information regarding medical risk factors for an elective CS was retrieved from the hospital’s birth record. Each risk factor was treated as a dichotomous variable depending on whether or not it was present during pregnancy. Potential risk factors were: (1) heart disease; (2) chronic hypertension; (3) chronic kidney disease; (4) asthma; (5) epilepsy; (6) rheumatoid arthritis; (7) diabetes; (8) gestational hypertension; (9) preeclampsia before week 34; (10) twins; (11) breech, breech/feet, or transverse lie; (12) large fetus (>4500 g); and (13) having previously delivered by CS. Medical risk was then coded as “1” (one or more risk factors present) or “0” (no risk factor present).

Furthermore, we assessed information on parity (nulliparous “0” or parous “1”) during pregnancy week 17. Age at delivery and maternal education were obtained from the hospital’s birth records. Educational level was coded as “1” (more than 12 years of education) and “0” (12 or fewer years of education).

### Statistical analyses

The analyses were conducted in three steps. First, descriptive analyses were carried out in order to obtain the total means and standard deviations (SD) of all variables. Second, on the basis of their preference and actual mode of delivery, we assigned the women to one of four groups: *Match 1* (no preference for CS, no elective CS), *Match 2* (preference for CS, elective CS), *Mismatch 1* (no preference for CS, elective CS), and *Mismatch 2* (preference for CS, no elective CS). By means of an analysis of variance (ANOVA), we examined whether the level of post-traumatic stress symptoms following childbirth differed significantly between these groups. Likewise, ANOVA were conducted to examine group differences for the putative risk factors. Bonferroni post hoc tests were conducted when such differences were detected. Third, all potential risk factors were correlated with post-traumatic stress symptoms following childbirth. The putative risk factors that were significantly correlated with post-traumatic stress symptoms were then entered as covariates in an analysis of covariance (ANCOVA) to examine if their inclusion was associated with a reduced group effect on post-traumatic stress symptoms following childbirth. To start with, each risk factor was included one by one. Next, related risk factors – i.e. (a) psychological factors, (b) personality, (c) somatic and demographic factors – were included simultaneously as covariates in one analysis each. Finally, all risk factors that were statistically significant in the previous model were included together in one ANCOVA.

Due to potential non-normal distribution of post-traumatic stress symptoms, additional ANCOVA were performed with log transformed values of PTSD, and the results of these analyses were compared with those from the initial analyses. Missing values on the psychometric scales were substituted with the mean of each case if the number of missing items was ≤ 20% (for the Mini-IPIP scales, maximum one out of four items); otherwise, they were excluded from the analyses. Our final sample consisted of 1,700 women. The statistical package IBM SPSS Statistics 20 was used for all analyses.

## Results

Mean maternal age at delivery was 31.2 years (SD 4.6 years, range 18.8–45.4 years); 5.2% of the women had previously delivered by a CS, 18.4% had a medical risk for an elective CS (including previous CS) and 47.2% were first-time mothers. Most of the women were married or cohabitating (97.8%) and did not smoke at the time of delivery (96.1%). Sixty-eight percent had more than 12 years of education. Compared with the national data from the Medical Birth Registry of Norway from 2009, the women in the study were less likely to be smokers (3.9% vs. 8.2% at the time of delivery) and, were slightly older (mean age of 31.2 years vs. 29.7 years), and there were fewer single women in the study (2.3% vs. 9.1%) [16].

Table 1 shows the means and SDs for all variables. In our sample, 6.0% had scores above 19 on the Impact of Event Scale indicating clinically significant levels of stress, and 1.8% scored above 34, indicating that a PTSD condition was likely [19]. The average score for post-traumatic stress symptoms following childbirth was 6.70 (SD = 7.99) (Table 1). The mean score for the subscale intrusion was 4.28 (SD = 4.85) and for the subscale avoidance 2.35 (SD = 3.89). Of the women in the sample, 9.7% would have preferred to have a CS, and 5.6% actually delivered by elective CS. Most women (N = 1,493; 87.8%) belonged to the Match 1 group (no preference for CS, no elective CS). Match 2 (preference for CS, elective CS) comprised 53 women (3.1%). Mismatch 1 (no preference for CS, elective CS) and Mismatch 2 (preference for CS, no elective CS) comprised 42 (2.5%) and 112 (6.6%) women, respectively. The four match/mismatch groups differed substantially in terms of their medical risk (F = 93.16, p < 0.001) and fear of childbirth (F = 37.82, p < 0.001) (Table 1). Fear of childbirth was particularly high in Match 2 and Mismatch 2, groups comprising women who had a preference for CS, whereas medical risk was particularly high in Match 2 and Mismatch 1, comprising women who actually delivered by elective CS.

**Table 1:** Characteristics of women in the match/mismatch groups reported in mean scores (Standard deviations)

**Variable**	**Total**	**Match 1**	**Match 2**	**Mismatch 1**	**Mismatch**	**F**	**Post hoc**^**b**^
**(Time point of measurement)**		**No preference, no ElCS**^**a**^	**Preference, ElCS**	**No preference, ElCS**	**2 preference, no ElCS**		
		***(N = 1,493)***	***(N = 53)***	***(N = 42)***	***(N = 112)***		
	**Mean**	**Mean**	**Mean**	**Mean**	**Mean**		
	**(SD)**	**(SD)**	**(SD)**	**(SD)**	**(SD)**		
PTSD symptoms (8 weeks postpartum)	6.70 (7.99)	6.32 (7.49)	7.70 (9.11)	8.03 (9.49)	10.83 (11.44)	11.96^***^	1-4^***^
*Psychological Factors*							
Prior PTSD (pregnancy week 17)	0.24 (0.77)	0.20 (0.66)	0.53 (1.51)	0.50 (1.21)	0.54 (1.17)	11.38^***^	1-2^*^, 1-4^***^
Prior Sexual Abuse^c^ (8 weeks postpartum)	0.16 (0.37)	0.16 (0.36)	0.19 (0.40)	0.19 (0.40)	0.27 (0.44)	3.35^*^	1-4^*^
Fear of childbirth (pregnancy week 32)	56.41 (19.70)	54.66 (18.16)	69.07 (30.40)	61.23 (19.08)	72.02 (23.78)	37.82^***^	1-2^***^, 1-4^***^, 3-4^*^
Depression (pregnancy week 32)	4.91 (4.17)	4.72 (3.99)	6.54 (4.78)	5.50 (5.42)	6.44 (5.10)	9.18^***^	1-2^**^, 1-4^***^
Anxiety (pregnancy week 32)	12.78 (3.11)	12.66 (3.01)	13.55 (3.54)	13.14 (3.48)	13.93 (3.83)	7.23^***^	1-4^***^
*Personality (pregnancy week 17)*							
Neuroticism	10.92 (3.12)	10.81 (3.08)	10.92 (3.57)	11.17 (3.09)	12.26 (3.18)	7.68^***^	1-4^***^
Conscientiousness	16.17 (2.57)	16.18 (2.55)	15.82 (2.97)	16.50 (2.51)	16.06 (2.54)	0.63	
Extraversion	14.12 (3.14)	14.13 (3.16)	14.50 (2.57)	14.00 (3.66)	13.93 (3.02)	0.42	
Agreeableness	17.49 (2.00)	17.50 (1.99)	17.34 (2.29)	18.08 (1.64)	17.16 (2.18)	2.34	
Imagination	13.81 (2.56)	13.80 (2.57)	14.26 (2.55)	13.48 (2.70)	13.82 (2.46)	0.80	
*Somatic & Demographic Factors*							
Medical risk (perinatally)	0.18 (0.39)	0.15 (0.35)	0.66 (0.48)	0.90 (0.30)	0.21 (0.41)	93.16^***^	1-2^***^, 1-3^***^, 2-3^**^, 2-4^***^, 3-4^***^
Parity (pregnancy week 17)	0.53 (0.50)	0.53 (0.50)	0.79 (0.41)	0.57 (0.50)	0.40 (0.49)	7.53^***^	1-2^**^, 2-4^***^
Age (perinatally)	31.19 (4.59)	31.12 (4.56)	32.85 (4.77)	32.68 (4.16)	30.72 (4.93)	4.31^**^	1-2^*^, 2-4^*^
Education (perinatally)	0.68 (0.47)	0.69 (0.46)	0.64 (0.48)	0.57 (0.50)	0.56 (0.50)	3.66^*^	1-4^*^

^a^Elective Cesarean Section.

^b^Bonferroni post-hoc tests are used ; 1–2 = significant differences between Match 1 and Match 2, 1–3 = significant differences between Match 1 and Mismatch 1, 1–4 = significant differences between Match 1 and Mismatch 2, 2–3 = significant differences between Match 2 and Mismatch 1, 2–4 = significant differences between Match 2 and Mismatch 2, 3–4 = significant differences between Mismatch 1 and Mismatch 2.

^*^p < .05, ^**^p < .01, ^***^p < .001.

^c^Prior sexual abuse, medical risk, parity and education are dichotomous variables, means are therefore indicating proportions.

Regarding post-traumatic stress symptoms, we found a significant interaction effect between preference and actual mode of delivery (F = 7.15, p = .008), and in examining differences for all four match/mismatch groups, ANOVA yielded significant overall group differences (F = 11.96, p < 0.001). However, although the Mismatch 1 and Mismatch 2 groups had a tendency toward higher levels of post-traumatic stress symptoms than the Match 1 and Match 2 groups, Bonferroni post-hoc tests found significant differences only between Match 1 (no preference for CS, no elective CS) and Mismatch 2 (preference for CS, no elective CS) (Table 1).

The correlations with putative risk factors showed that post-traumatic stress symptoms were most strongly related to symptoms of depression and anxiety, but fear of childbirth and neuroticism also showed considerable correlations with such symptoms (Table 2). When risk factors were included one by one as covariates in ANCOVA, the inclusion of fear of childbirth had the greatest potency in explaining group differences in post-traumatic stress symptoms, as the F-value of the group effect was reduced from 11.96 to 4.29. Symptoms of depression and anxiety (reduced to 7.35 and 7.61), and to some degree neuroticism and prior PTSD (reduced to 8.46 and 8.79), also decreased the F-value. However, even though the F-value was substantially reduced by some of the covariates, the overall group effect and the difference in the level of post-traumatic stress symptoms between Match 1 and Mismatch 2 still remained significant, as indicated by post hoc tests. When all risk factors were entered blockwise, all risk factors remained statistically significant (Table 3). However, the block with the psychological risk factors showed the greatest potency in explaining group differences, compared to personality variables and somatic and demographic factors. Including the psychological factors reduced the F-value to 3.17, but the group differences remained significant (Table 3). Within the block with psychological risk factors, fear of childbirth was the most important covariate (F = 32.76, $\eta_{p}^{2}$ = 0.019), followed by symptoms of depression (F = 26.05, $\eta_{p}^{2}$ = 0.015) and anxiety (F = 22.77, $\eta_{p}^{2}$ = 0.013). Within the block comprising personality, neuroticism was the most important covariate (F = 78.73, $\eta_{p}^{2}$ = 0.044), and within the block with somatic and demographic factors, parity had the largest effect (F = 20.41, $\eta_{p}^{2}$ = 0.012) (Table 3).

**Table 2:** Correlations between posttraumatic stress symptoms following childbirth and all putative risk factors

	**1.**	**2.**	**3.**	**4.**	**5.**	**6.**	**7.**	**8.**	**9.**	**10.**	**11.**	**12.**	**13.**	**14.**
1. PTSD symptoms (8 weeks postpartum)	1													
2. Prior PTSD (pregnancy week 17)	0.19^***^	1												
3. Prior Sexual Abuse^a^ (8 weeks postpartum)	0.13^***^	0.24^***^	1											
4. Fear of childbirth (pregnancy week 32)	0.28^***^	0.11^***^	0.08^**^	1										
5. Depression (pregnancy week 32)	0.35^***^	0.28^***^	0.18^***^	0.40^***^	1									
6. Anxiety (pregnancy week 32)	0.33^***^	0.33^***^	0.16^**^	0.31^***^	0.68^***^	1								
7. Neuroticism (pregnancy week 17)	0.23^***^	0.22^***^	0.12^***^	0.29^***^	0.48^***^	0.41^***^	1							
8. Conscientiousness (pregnancy week 17)	−0.11^***^	−0.09^***^	−0.07^**^	−0.16^***^	−0.15^***^	−0.15^***^	−0.13^***^	1						
9. Extraversion (pregnancy week 17)	−0.02	−0.03	−0.03	−0.14^***^	−0.11^***^	−0.12^***^	−0.12^***^	0.16^***^	1					
10. Agreeableness (pregnancy week 17)	0.00	0.03	0.03	−0.12^***^	−0.01	0.01	−0.05	0.21^***^	0.31^***^	1				
11. Imagination (pregnancy week 17)	0.01	0.02	0.05^*^	−0.04	0.03	0.04	0.00	0.02	0.25^***^	0.28^***^	1			
12. Medical risk (perinatally)	0.04	0.09^***^	0.09^***^	0.02	0.04	0.06^*^	0.02	−0.03	−0.01	0.01	0.00	1		
13. Parity (pregnancy week 17)	−0.15^***^	−0.04	−0.05	−0.14^***^	0.03	−0.01	−0.02	0.03	−0.04	−0.04	0.00	0.08^**^	1	
14. Age (perinatally)	−0.12^***^	−0.13^***^	−0.02	0.00	−0.07^**^	−0.15^***^	−0.11^***^	0.11^***^	−0.01	−0.02	0.08^**^	0.06^**^	0.40^***^	1
15. Education (perinatally)	−0.08^**^	−0.11^***^	−0.09^***^	−0.01	−0.09^***^	−0.13^***^	−0.13^***^	0.03	0.05^*^	0.06^*^	0.13^***^	−0.05	−0.01	0.26^***^

^*^p < .05, ^**^p < .01, ^***^p < .001.

^a^Prior sexual abuse, medical risk, parity and education are dichotomous variables.

**Table 3:** Adjusted means of posttraumatic stress symptoms following childbirth in four different match/mismatch groups

	***Covariates***		***Adjusted means and difference test of posttraumantic stress symptoms***					
**Variable**	**η**_**p**_^**2**^	**F**	**Match 1**	**Match 2**	**Mismatch 1**	**Mismatch 2**	**F**	**Post hoc**^**b**^
**(Time point of measurement)**			**No preference, no ElCS**^**a**^	**Preference, ElCS**	**No preference, ElCS**	**Preference, no ElCS**		
			*Model 1 (covariates from each block included simultaneously)*					
*Psychological Factors*			6.56	5.97	7.25	8.73	3.17^*^	1-4^*^
Prior PTSD (pregnancy week 17)	0.004	6.33^*^						
Prior Sexual Abuse^c^ (8 weeks postpartum)	0.002	3.89^*^						
Fear of childbirth (pregnancy week 32)	0.019	32.76^***^						
Depression (pregnancy week 32)	0.015	26.05^***^						
Anxiety (pregnancy week 32)	0.013	22.77^***^						
*Personality*			6.38	7.61	7.97	10.08	8.55^***^	1-4^***^
Neuroticism (pregnancy week 17)	0.044	78.73^***^						
Conscientiousness (pregnancy week 17)	0.006	10.22^**^						
*Somatic & Demographic Factors*			6.32	8.35	8.19	10.43	10.88^***^	1-4^***^
Parity (pregnancy week 17)	0.012	20.41^***^						
Age (perinatally)	0.003	5.77^*^						
Education (perinatally)	0.002	4.24^*^						
			*Model 2 (all statistically significant covariates included simultaneously)*					
*All covariates*			6.54	6.69	7.46	8.56	2.66^*^	1-4^*^
Prior PTSD (pregnancy week 17)	0.002	3.98^*^						
Prior Sexual Abuse (8 weeks postpartum)	0.002	2.87						
Fear of childbirth (pregnancy week 32)	0.012	20.85^***^						
Depression (pregnancy week 32)	0.016	26.55^***^						
Anxiety (pregnancy week 32)	0.010	17.74^***^						
Neuroticism (pregnancy week 17)	0.001	1.80						
Conscientiousness (pregnancy week 17)	0.001	1.11						
Parity (pregnancy week 17)	0.013	22.13^***^						
Age (perinatally)	0.001	1.17						
Education (perinatally)	0.000	0.69						

^a^Elective Cesarean Section.

^b^Bonferroni post-hoc tests are used ; 1–4 = significant differences between Match 1 and Mismatch 2.

^*^p < .05, ^**^p < .01, ^***^p < .001.

^c^Prior sexual abuse, medical risk, parity and education are dichotomous variables, means are therefore indicating proportions.

When all covariates were entered simultaneously in one ANCOVA (they were all statistically significant in the previous model and thus included), the F-value was further reduced to 2.66; however, the group differences remained significant (Table 3). In this model, symptoms of depression (F = 26.55,$\eta_{p}^{2}$ = 0.016), parity (F = 22.13, $\eta_{p}^{2}$ = 0.013), fear of childbirth (F = 20.85, $\eta_{p}^{2}$ = 0.012) and symptoms of anxiety (F = 17.74, $\eta_{p}^{2}$ = 0.010) were the most important covariates. The other risk factors (except prior PTSD, F = 3.98, $\eta_{p}^{2}$ = 0.002) were no longer statistically significant (Table 3).

All ANCOVA were, furthermore, repeated with log transformed scores of the Impact of Event Scale. Besides minor changes in the F-values, the results were very similar to the initial analyses without transformation.

## Discussion

To our knowledge, this prospective cohort study is the first to examine whether a *mismatch* between preferred and actual mode of delivery increases the risk of developing post-traumatic stress symptoms following childbirth. The key findings are that (a) women who preferred delivery by CS, but whose actual mode of delivery was vaginal, had a higher level of post-traumatic stress symptoms, and that (b) psychological factors such as fear of childbirth, symptoms of depression, and anxiety were particularly potent risk factors that could explain parts of this effect.

Hence, the association between such mismatch and posttraumatic stress symptoms could be partly attributed to psychological vulnerability, particularly fear of childbirth. Therefore, even though a normal vaginal delivery constitutes a comparatively mild stressor, under certain circumstances and combined with psychological vulnerability it may be severe enough to produce symptoms of post-traumatic stress following childbirth. Söderquist and colleagues also found that among primiparous women who had a vaginal delivery, one out of two women with post-traumatic stress symptoms following childbirth had a history of psychiatric/psychological counseling [7]. Psychologically vulnerable women and women with a fear of childbirth may fear the labor process itself, because they perceive it as uncontrollable, unfamiliar, and frightening [30]. This may explain why women with a fear of childbirth would prefer a CS [31]. However, if these women have to deliver vaginally while their fears remain unacknowledged, they may feel over-ridden and may perceive that their physical integrity is threatened in a very intimate way, which may even result in post-traumatic stress symptoms.

Nulliparous women were at a higher risk of developing traumatic stress compared to parous women, a finding that is consistent with previous research [32]. Moreover, in addition to perinatal psychological factors, parity and a prior experience of PTSD could explain a small part of the group differences. Hence, not only current psychological vulnerability, but also a woman’s psychological and obstetric history seems to be important, regarding birth expectations and traumatic stress following birth.

We found an effect only for women who preferred a CS but had a vaginal delivery, and not for those women who preferred a vaginal delivery but had an elective CS. This finding is in line with another study that showed a heightened risk for post-traumatic stress symptoms after an emergency CS but not after an elective CS [33]. Usually, delivery by elective CS is a very controlled and quick procedure. Also, unless a CS is conducted upon maternal request (which is not the case if the women prefer a vaginal delivery), women are delivered by elective CS for medical reasons [17]. These circumstances may make it easier for the women to accept and cope with an elective CS even though they would have preferred a vaginal delivery.

As we expected, neither of the two Match groups was associated with a higher level of post-traumatic stress symptoms. In contrast to our findings, Karlström and colleagues found that women who preferred and actually delivered by an elective CS (i.e. “Match 2”) were less satisfied with both their antenatal care and their birth experience compared to women who preferred to give birth vaginally and actually had a spontaneous vaginal birth. However, post-traumatic stress symptoms, which are more severe than mere dissatisfaction, were not assessed [13].

### Clinical implications

From an obstetric point of view, a vaginal delivery is usually considered the best option in the absence of medical indications, because delivery by CS is associated with increased infant and maternal risks. In addition, a CS has implications for future pregnancies and resource implications for health services [17]. Still, some women, e.g. those with an intense fear of childbirth, wish for a CS despite being physically capable of delivering vaginally. Our results suggest that those women who prefer a delivery by CS but actually deliver vaginally have an increased risk for post-traumatic stress symptoms following childbirth.

To avoid potential traumatization, emphasizing the risk of CS does not seem to be sufficient to persuade a woman to deliver vaginally. Rather, fear of childbirth and a maternal request for CS should be taken seriously and treated adequately. Studies have shown that at least half of the women can, after treatment (e.g. crisis-oriented intervention, intensive therapy or group psychoeducation combined with relaxation exercises), prepare for a normal vaginal delivery [34-37]. One study also showed that they remained pleased with their choice afterward [36].

Still, not all women change their minds. In a white paper, the Norwegian Ministry of Health stresses that an elective CS should be medically indicated; nevertheless, attentiveness to patient preferences should be a part of the calculation underlying the decision [38]. Other public health systems such as those in the UK are even more liberal regarding CS on maternal request, and acknowledge fear of childbirth as a valid indication [39]. In fact, it is uncertain whether women forced to undergo a vaginal delivery against their wishes actually end up with better outcomes than if they had a planned CS. For instance, in a another study by our team, we found that labour duration was significantly longer in women with fear of childbirth [40]. Also, we found that fear of childbirth increased the risk of a negative subjective birth experience which in turn may lead to post-traumatic stress symptoms [9].

Whether or not a woman has the right to choose her mode of delivery remains an ongoing debate. However, in extreme cases of fear of childbirth, CS may be appropriate if counseling and support during pregnancy have not been effective and if there is considerable risk to the woman’s health and wellbeing [41].

### Limitations

Readers should also note some limitations of our findings. First, although the entire study sample consisted of 1,700 women, some of the groups were rather small; particularly, Match 2 (N = 53) and Mismatch 1 (N = 42), which did not differ significantly from any other group. We may have been able to find more group differences given a larger sample size in these two groups.

Second, we did not assess the reasons for prefering a specific mode of delivery. For instance, some women may have wished for a CS because of a pre-existing medical condition or complication, and not because of fear of childbirth. Thus, each of the four groups might be somewhat heterogeneous due to differing motivations for preferred mode of delivery. Further, we do not know whether the women’s preference for mode of delivery remained the same from pregnancy week 32 up to the actual delivery or whether they shared their preferences with the health care staff. In a similar vein, we do not know whether the women from Mismatch 2 were forced to deliver vaginally or whether they eventually changed their mind.

Finally, the generalizability of the results is limited because only Norwegian-speaking women were included. This resulted in a relatively homogeneous, mainly Caucasian sample. Different results might be obtained for other ethnic groups. Furthermore, with regard to socio-demographic characteristics, there is reason to believe that there is a slight social gradient associated with participation. However, it is important to bear in mind that selection bias does not necessarily influence the results when associations between variables are investigated [42].

## Conclusion

Post-traumatic stress symptoms following childbirth are an important women’s health issue. The results of this study suggest an increased level of post-traumatic stress symptoms for women who experience a mismatch between their preferred and actual mode of delivery, i.e. for women who prefer a delivery by CS but who actually deliver vaginally. Particularly in psychologically vulnerable women who suffer from fear of childbirth or symptoms of depression or anxiety, this mismatch may threaten their physical integrity, and in turn result in post-traumatic stress symptoms following childbirth. Women with a fear of childbirth may prefer a CS even though a vaginal delivery is usually the best option in the absence of medical indications. Because psychological and obstetric factors could explain only a relatively small amount of the variance in post-traumatic stress symptoms, future studies should examine whether a mismatch in the expectations and the experience of the psycho-social environment during birth (e.g. how maternity staff treat women, how much control they perceive they have) might also increase the risk of post-traumatic stress.

At any rate, to avoid potential traumatization, fear of childbirth and maternal requests for CS should be taken seriously and treated adequately, for instance with the help of group psychoeducation or crisis-oriented counseling.

## Competing interests

The authors declare that they have no competing interests.

## Authors’ contributions

SGN designed the study, performed the statistical analyses, and drafted the manuscript. TvS participated in the design of the study, helped with the statistical analyses, and helped to draft the manuscript. CK and LT participated in the design of the study and were involved in revising the manuscript content. TBS was involved in the organization of the study, the data collection and the writing of the manuscript. MEG conceived of the ABC study, participated in the design of the present study, and helped to revise the manuscript content. All authors read and approved the final manuscript.

## Pre-publication history

The pre-publication history for this paper can be accessed here:

http://www.biomedcentral.com/1471-2393/14/191/prepub
